# Kinetics of Decelerated Melting

**DOI:** 10.1002/advs.201700850

**Published:** 2018-03-01

**Authors:** Lothar Wondraczek, Zhiwen Pan, Theresia Palenta, Andreas Erlebach, Scott T. Misture, Marek Sierka, Matthieu Micoulaut, Uwe Hoppe, Joachim Deubener, G. Neville Greaves

**Affiliations:** ^1^ Otto Schott Institute of Materials Research University of Jena 07743 Jena Germany; ^2^ Center of Energy and Environmental Chemistry University of Jena 07743 Jena Germany; ^3^ Inamori School of Engineering Alfred University Alfred NY 14802 USA; ^4^ Physique Théorique de la Matière Condensée Paris Sorbonne Universités – UPMC 75252 Paris France; ^5^ Institute of Physics Rostock University 18059 Rostock Germany; ^6^ Institute of Non‐Metallic Materials Clausthal University of Technology 38678 Clausthal‐Zellerfeld Germany; ^7^ Department of Materials Science and Metallurgy University of Cambridge CB3 0FS Cambridge UK; ^8^ Department of Physics Aberystwyth University SY23 3BZ Aberystwyth UK

**Keywords:** kinetics, melting, metal–organic frameworks, simulations, zeolites

## Abstract

Melting presents one of the most prominent phenomena in condensed matter science. Its microscopic understanding, however, is still fragmented, ranging from simplistic theory to the observation of melting point depressions. Here, a multimethod experimental approach is combined with computational simulation to study the microscopic mechanism of melting between these two extremes. Crystalline structures are exploited in which melting occurs into a metastable liquid close to its glass transition temperature. The associated sluggish dynamics concur with real‐time observation of homogeneous melting. In‐depth information on the structural signature is obtained from various independent spectroscopic and scattering methods, revealing a step‐wise nature of the transition before reaching the liquid state. A kinetic model is derived in which the first reaction step is promoted by local instability events, and the second is driven by diffusive mobility. Computational simulation provides further confirmation for the sequential reaction steps and for the details of the associated structural dynamics. The successful quantitative modeling of the low‐temperature decelerated melting of zeolite crystals, reconciling homogeneous with heterogeneous processes, should serve as a platform for understanding the inherent instability of other zeolitic structures, as well as the prolific and more complex nanoporous metal–organic frameworks.

## 1 Introduction

As one of the most dramatically visible phase transitions, melting processes are of fundamental importance across all fields of condensed matter science. Regarding the microscopic mechanism which leads to the transformation of a solid into a liquid, our understanding rests largely on the early works of Lindemann^1^ and Gilvarry.^2^ These led to a criterion of melting, whereby structural instability occurs when the amplitude of atomic thermal vibrations exceeds ≈10% of the interatomic distance. Based on Einstein's model of harmonic atomic oscillation and heat capacity,^3^ the theory successfully predicts melting temperatures of close‐packed solids, but has serious shortcomings for less‐dense materials in only considering the average atom reduced to a simple cubic lattice, and in underestimating the vibrational dynamics.^4^ The vast range of network structures (with free volume and complex dynamic parameters like cooperative motion, bond rupture, and interactions between solid and liquid phases) do not follow the Lindemann criterion.

A major conundrum has been the interplay between superheating and surface melting, or, more generally, homogeneous and heterogeneous melting. Following Frenkel's dismissive statement^5^ that superheating is generally avoided as crystal melting always begins at the surface, there has now been strong evidence for the apparent universality of surface melting^6^ and its prominent role in diverse fields, including materials science, geophysics, biological chemistry, and climatology.^7^, ^8^, ^9^, ^10^ On the other hand, direct observations of bulk melting have also become possible through advances in molecular dynamic (MD) simulation,^11^, ^12^, ^13^ colloidal processing,^14^, ^15^, ^16^ and in combinations of rapid heating and high‐resolution imaging.^17^, ^18^, ^19^

The lack of insight into bulk melting is exacerbated by the very short timescale within which crystals transform into the molten state.^17^, ^18^ The sluggish but tailorable dynamics of colloidal materials has been exploited as a physical model for bypassing this problem.^20^, ^21^ Regarding observation on the molecular scale, we now argue that a dramatically lower melt mobility, such as encountered when the viscosity of the material at its liquidus temperature is extremely high, presents a kinetic barrier to crystal melting—the energy barrier for nucleation of the melt no longer being dominated by the surface energy. In this event, the material initially retains the crystalline topology, with the time dependence of melting determined by the melt viscosity.

For more than a century,^22^ classical nucleation theory has been employed to describe melting processes, whereby embryonic regions of liquid mobility occur as a result of random thermal fluctuations. On reaching the critical nucleation size, these grow irreversibly until the solid transforms into a liquid.^23^ Criticality may occur for just a few atoms, significantly lower than predicted theoretically.^12^ Furthermore, instead of a single reaction barrier, rare event sampling by MD simulations has revealed a multitude of microscopic reaction pathways occurring in bulk melting.^13^

There are some archetypal melts, such as SiO_2_ or albite (NaAlSi_3_O_8_), in which the viscosity at the liquidus, η(*T*_m_) is comparably high. For silica η(*T*_m_) ≈ 10^5.46^ Pa s^24^ and for albite exhibiting pronounced surface pre‐melting, η(*T*_m_) ≈ 10^6.3^ Pa s.^25^ Both silica and albite have excellent glass‐forming ability with shortened supercooled ranges, *T*_g_/*T*_m_ exceeding 2/3.^38^ However, the corresponding maximum shear relaxation times for silica and albite are still low, 9.6 and 66 µs, respectively, suggesting that fluid dynamics would not present a major obstacle to bulk melting even in these very strong liquids. Indeed, under moderate heating rates, most crystals rapidly melt at their liquidus temperature^26^ and it is often assumed that this is facilitated by the surface dynamics which reduce the energy barrier of melt nucleation.^27^ Superheating per se is usually only observed in highly confined geometry or during ultrafast heating.^28^

**Figure 1:**
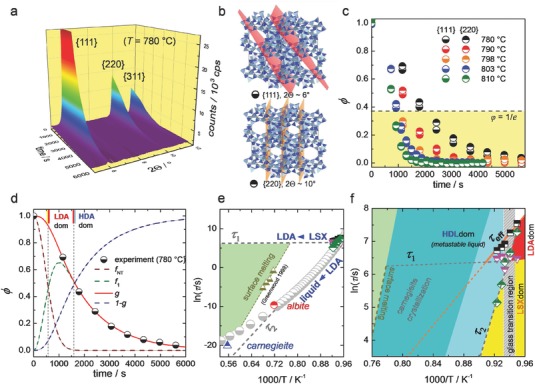
Decelerated melting of LSX (Na_19_K_77_Al_96_Si_96_O_384_). a) Exemplary contour plot of in situ X‐ray diffraction data recorded during isothermal treatment at 780 °C. b) Structural location of the lattice planes used for evaluation. c) Crystallinity loss φ constructed from (a) using Equation (1). d) φ data fit to the kinetic model (Equation (2)), displaying individual fractions of LSX and LDA for melting at 780 °C. e,f) Arrhenius scaling of reaction times τ_1_ and τ_2_, the melt relaxation time of albite and carnegieite at *T*_m_, melt viscosity (gray dots), and data extracted from pre‐melting of albite showing surface melting of a single lattice plane,^34^ with extrapolations of Arrhenius fits (dashed gray lines). f) Onset of melting, highlighting the different reaction timescales and the dominant phases LSX, LDA, and HDL (liquid) in time–temperature space. The darker‐shaded area in (f) indicates the experimental occurrence of carnegieite recrystallization according to ref. 32.

**Figure 2:**
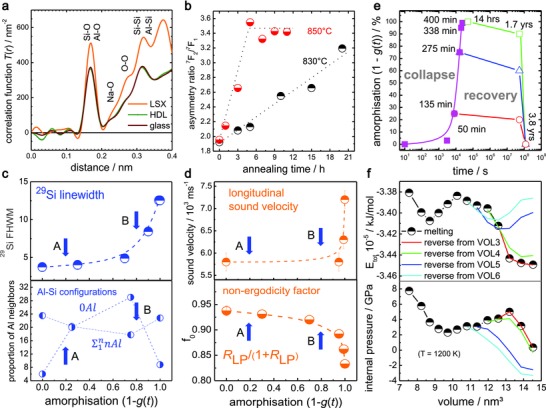
Structural signature of decelerated faujasite melting. a) Structural equivalence of quenched HDL and conventional glass from synchrotron XRD. b) Asymmetry ratio of Eu^3+^ photoluminescence with melting. c) Ex situ increase in ^29^Si NMR linewidths with melting (top) and converse changes in the proportions of Si‐Al neighbors (bottom). d) Ex situ room temperature IXS with melting revealing a nonlinear decrease of the nonergodicity factor *f*_0_ (bottom) and an increase in the longitudinal sound velocity *V*_L_ (top). Arrows in (c) and (d) indicate the order–order LSX‐LDA (A) and the order–disorder LDA‐HDL transitions (B). e) Recovery of faujasite crystallinity over periods of years for material initially amorphized to 25, 75, 99, and 100%. f) Experimental (top) and computational data (bottom) showing the reversibility of the first reaction step of zeo‐LDA and LSX‐LDA, respectively. Data in (a) and (b) are from LSX and LSX Eu, respectively, data in (c)–(e) are from Na zeolite, and (f) are for silicalite and MD simulations—all exhibiting identical faujasite topology. Lines are to guide the eye.

**Figure 3:**
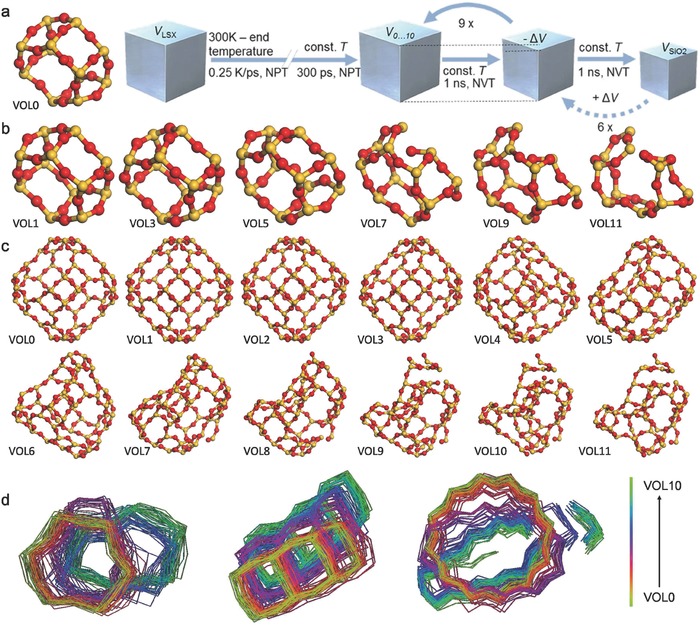
Molecular dynamics simulation of silicalite amorphization. a) Schematic of the computational procedure. b–d) Detailed trajectories of the reaction progress on different superstructural scales, where each volume step represents a density increase of ≈0.089 g cm^−3^. Yellow and red balls in (b) and (c) represent silicon and oxygen ions, respectively. In (d), the evolution of the three fundamental building blocks is depicted, that is, a six‐membered ring, three edge‐sharing four‐membered rings, and the 12‐membered ring forming the faujasite cage.

We now consider the melting of materials with large free volume, for example, mesoporous organic, inorganic, or hybrid frameworks, in which melting involves a metastable liquid, similar to the decompression‐melting of crystalline bismuth.^29^ Due to the very high free volume of the precursor phase, structural collapse occurs close to the glass transition temperature of the corresponding liquid,^30^, ^31^ which causes deceleration on the scale of minutes. In situ X‐ray diffraction (XRD) studies of isothermally annealed low‐silica (Na, K) zeolite X (LSX) (Na_19_K_77_Al_96_Si_96_O_384_) reveal that the kinetics of this reaction are nontrivial, suggesting the presence of a two‐step process of structural distortion and diffusive motion, further quantified using a simple kinetic model (**Figure** **1**). Nuclear magnetic resonance (NMR) and inelastic X‐ray scattering (IXS) experiments have also been conducted, on Na zeolite Y (Na_59_Al_59_Si_133_O_384_) which together with LSX belongs to the faujasite family, but with differing Al/Si ratios, to look for complementarity from common topology (**Figure** **2**). Results are correlated with MD simulations of faujasite silicalite (SiO_2_) that confirm the two reaction steps and their structural signature (**Figure** **3**). The model allows for reversibility, whereby over large timescales the faujasite structure might be restored as observed experimentally. These findings highlight the distinct roles of local structural instability and diffusive motion which act in concert during the transformation of a solid into a liquid, hence reconciling our understanding of bulk and surface melting within a single model.

## 2 Results

### 2.1 Decelerated Melting in Low‐Density Crystals

Figure 1a reveals how the melting of the faujasite cage structure occurs continuously in a timescale of 10^3^ s for temperatures around 780–803 °C. From in situ X‐ray diffractograms, the time‐dependent loss of crystallinity is evaluated on individual lattice planes (Figure 1b,c), following normalization employing the general Kohlrausch–Williams–Watt (KWW) function

(1) $$\varphi (t)\;=\;\frac{A(t)-A_{\infty }}{A_{0}-A_{\infty }}\;=\;\exp\left[-{\left(\frac{t}{\tau }\right)}^{\;\beta }\right]$$

Here, ϕ(*t*) is the reaction progress, *A*(*t*), *A*_∞_, and *A*_0_ are the diffraction peak areas at time *t*, extrapolated to infinite time, and at the start of the reaction, respectively, τ presents the reaction timescale, and β is the KWW exponent.

Initially fitting the data in Figure 1c to Equation (1) requires values for β exceeding unity, clearly opposing the usual stretched exponential relaxation function (β < 1) which is associated with the dynamics of metastable liquids.^33^ Furthermore, there is no signature of anisotropic instability, given that the decay functions for different lattice planes are essentially identical.

To understand the experimental observation of compressed exponential kinetics, we now propose a simple model which captures the observed signature of melting. This starts by assuming that the overall transition proceeds in two steps, that is, the transformation of the ordered crystal (LSX) into a distorted low‐density phase (LDA) and, subsequently, a high‐density liquid (HDL). It is furthermore assumed that the first reaction is governed by short‐range dynamics such as ballistic motion or cage‐rattling between sets of particles. These assumptions will be confirmed later from structural studies and atomistic simulation.

A normal (Gaussian) distribution function is taken for the probability of local instability, in which for the first reaction step β_LSX‐LDA_ = 2.0. For the reaction of final melting, the kinetic barrier is governed by longer‐ranging transport, usually modeled with β_LDA‐HDL_ < 1.0. However, the origin of the stretched exponential relaxation in melt dynamics lies with integration across a large ensemble of local states. The reaction of LDA→HDL occurs on length scales which do not reasonably allow for such integration, so that we assume a finite number of involved states^12^ with β_LDA‐HDL_ = 1.0.

The experimental data are now framed within this model. Following a simple reaction rate scheme for sequential reactions (S1) (Supporting Information), the loss in crystallinity *g*(*t*) follows the function

(2) $$\begin{array}{l} g\left(t\right)\;=\;f_{\mathrm{NT}}\left(t\right)\;+\;f_{1}\left(t\right)\;=\;\{\frac{\sqrt{\pi }}{2}\frac{\tau_{1}}{\tau_{2}}\exp\left(-\frac{t}{\tau_{2}}\right)\exp\left({\left(\frac{\tau_{1}}{2\tau_{2}}\right)}^{2}\right)\\ \;\;\;\;\;\;\;\;\;\;\;\left[\mathrm{erf}\left(\frac{\tau_{1}}{2\tau_{2}}\right)\;-\;\mathrm{erf}\left(\frac{\tau_{1}}{2\tau_{2}}-\frac{t}{\tau_{1}}\right)\right]\}\;+\;\exp\left(-\frac{t}{\tau_{2}}\right) \end{array}$$

Here, *f*_NT_ represents the initial solid fraction, decreasing with LSX→LDA, while *f*_1_ represents the transient LDA phase before transforming into HDL. Equation (2) provides an excellent fit to the experimental data (Figure 1d).

In order to judge the observed reaction timescales τ_1_ and τ_2_ they are compared to the dynamics of viscous flow and surface pre‐melting. Details of this analysis are provided in Section S2 (Supporting Information). Figure 1e,f shows Arrhenius plots of the experimental data, including the reaction times extracted from classical surface melting studies of crystalline albite.^34^ Extrapolation of τ_2_ leads to the Maxwell relaxation time of viscous flow at the melting points of the high‐density crystals of albite and carnegieite. The slopes of τ_1_ and τ_2_ reflect, respectively, superstrong behavior in the low‐density superheated zone and fragile behavior in the supercooled zone.^30^

### 2.2 Phase Transitions Revealed in the Structural Signature of Melting

Synchrotron X‐ray diffraction confirms that the molecular structure of the final HDL phase is almost identical to that of a corresponding melt‐quenched glass (Figure 2a), providing evidence that the overall reaction from LSX to HDL leads to the same state as a regular melting process at the melting point *T*_m_. According to viscosity analyses, we have a kinetic glass transition temperature (η = 10^12^ Pa s) of 793 °C for the melt‐quenched glass (Equation (S2), Supporting Information). In all experiments, decelerated melting occurs close to this temperature, which can be compared with *T*_g_ of 791 °C for supercooled carnegieite where the viscosity is 10^12^ Pa s^35^ and *T*_m_ for crystalline carnegieite (NaAlSiO_4_) at 1526 °C, where the viscosity is 10^1.7^ Pa s.^35^

The absence of any low‐density intermediate phase of the sample post mortem (790 °C, 2 h) complies fully with the analytical model of the collapse kinetics. The transient LDA phase, however, can be detected through time‐resolved structural analyses^30^ using Eu^3+^ as a photoluminescent probe.^36^ Figure 2b reveals that there is a continuous increase in local asymmetry over 20 h of annealing at 830 °C well before any obvious variations appear in the XRD data (Figure S5, Supporting Information), whereas at 850 °C local distortion reaches a plateau after only 6 h with the loss of microscopic crystallinity occurring after 7 h.

In Figure 2, the progress of two‐step decelerated melting observed through ^29^Si NMR (c) and IXS experiments (d) is shown for Na zeolite Y. From NMR spectra, the 5 Si‐Al configurations are readily identified in the starting crystal and are still evident (Figure S6, Supporting Information) even when only 25% of Na zeolite Y remains, confirming that crystalline topology is retained in the initial LDA phase (c). Both phase transitions, LSX→LDA (A) and LDA→HDL (B) can be identified from the proportions of 5 Si‐Al configurations, viz., ^0Al^Q_4_ compared to the average ^nAl^Q_4_ (*n* = 1 to *n* = 4), each intersecting around 20 and 80% amorphization (1 − *g*(*t*)) and quantifying the two transitions predicted from XRD (Figure 1d). In particular, these data suggest that the LSX‐LDA transition is mediated by a slackening of Lowensteinian order, perhaps by tetrahedral switching in the superheated state, with diffusion in the supercooled state restoring order in the final LDA‐HDL transition. The two transitions complement developments in the intensities of the Rayleigh and Brillouin features from IXS (d). Notably the nonergodicity factor *f*_0_, which measures the departure from thermodynamic equilibrium,^37^, ^45^ obtained from the Landau Placzek ratio *R*_LP_ (Figure S7, Supporting Information), and the longitudinal speed of sound *V*_L_ behave nonlinearly in low‐temperature melting, both changing abruptly beyond 80% amorphization (Figure 2d), end values being very similar to vitreous silica at *T*_g_,^45^, ^46^ as is the molar volume (Figure S8, Supporting Information).

These structural findings for Na zeolite Y complement those for LSX, with similar annealing temperatures, confirming that the melting process relates to their common faujasite topology,^30^ rather than the nearest neighbor Lindemann criterion.^1^, ^2^ From earlier inelastic neutron scattering studies,^46^ librational and collective THz vibrations were identified as correlating with low‐temperature melting: librational dynamics with the LDA formation and collective dynamics with the LDA‐HDA transition (Figure S9, Supporting Information). Notably, collective modes reflected faujasite topological features until the transformation to HDA and its featureless boson peak, very similar to that of vitreous silica.^45^, ^46^ Finally, the reversibility of the zeo‐LDA order–order transition, which share common topologies (Figure 3b,c), and of the liquid–liquid LDA‐HDA order–disorder transition were revealed over protracted time scales at room temperature, monitored with ex situ XRD (1 − *g*(*t*)) (Figure 2e). These observations are compared with atomistic simulation of the reversibility of the total energy and the internal pressure, through successive volume changes (Figure 2f), as described below.

### 2.3 Computational Simulation

MD simulation of the isothermal collapse of the alkali‐free faujasite framework silicalite was performed through stepwise volume reduction, mimicking the collapse during low‐temperature melting (Figure 3a). This provides a clear view of the evolution of topological structure across the two reaction steps (Figure 3b–d). The major change in total energy occurs within the first third of volume reduction from the initial silicalite to vitreous silica (steps VOL2–VOL4 (Figure S10, Table S4, Supporting Information)). The simulation temperature was raised in parallel with volume changes, from 300 K first to 800 K, and then in steps of 100–1200 K (Figure 3a). This density–temperature sequence leads to the build‐up of internal pressure which reaches a maximum at VOL3, where there is a plateau in total energy followed by a decrease in internal pressure before reaching VOL6 (Figure 2f). The intermediate‐range structure signature resembles experimental observation (Figure 2a), where the transition from faujasite to LDA retains the ring configuration, but not the overall periodicity, until reaching around 80% of reaction progress. During HDL formation, the inter‐tetrahedral distances across four‐ and sixfold rings converge to a single asymmetric feature. In this process, the supercages begin to break up, signifying the final melting step (Figure 3d).

## 3 Discussion

Using a combination of experimental and theoretical techniques, we have elucidated the origins of low‐temperature melting by studying zeolite frameworks with common topology, where melting is decelerated at the glass transition to tractable timescales. Employing a simple kinetic model the unusual compressed exponential kinetics (β > 1) that characterize decelerated melting are accurately reproduced by a two‐stage reaction sequence: (1) an order–order superheated transition from the nanoporous (expanded) crystal to a low density intermediate phase (β = 2, τ_1_), followed by (2) an order–disorder transition where the aperiodic solid LDA phase melts heterogeneously to a final HDL phase (β = 1, τ_2_). The very different temperature dependencies of the two reaction times τ_1_ and τ_2_ (extrapolated across the supercooled regime) define boundaries for the two reactions, which subtend the viscosities of the classic glass formers carnegieite and albite and the zone of surface melting. At the glass transition, they define the distinct domains of zeolite, LDA and HDL in time–temperature space. Appealing to a range of NMR, IXS and INS experiments, the two reactions involved in decelerated melting have been independently identified. Finally, modeling predictions and experimental observations of the kinetics of decelerated melting have been tested against extensive MD simulations, where confirmation of the two‐stage process has been obtained from the developments in total energy and internal pressure. Moreover, by visualizing atomic trajectories associated with the classical subunits that define faujasite topology, we have been able to uniquely identify their retention in the homogeneous superheated zeolite‐LDA reaction, and their destruction in the heterogeneous LDA‐HDL melting reaction that follows.

This successful quantitative modeling of the low‐temperature melting of zeolite crystals, reconciling homogeneous with heterogeneous processes, should serve as a platform for understanding the inherent instability of other zeolite structures, as well as the prolific and more complex metal–organic frameworks,^39^ where collapse at the glass transition has recently been observed.^31^

## 4 Experimental Section

*LSX Zeolite, Ion Exchange, and Melt‐Quenched Glass Synthesis*: Synthesis of the LSX followed the recommended procedure of the International Zeolite Commission (IZA^40^). For this, reagent grade chemicals sodium aluminate, potassium hydroxide, sodium hydroxide, and sodium silicate (water glass), and double‐distilled water were employed, resulting in a white crystalline powder which was filtered washed, and finally dried at 100 °C (**Table** **1**). Between experiments, all powders were stored in dry desiccators.

**Table 1:** Analyzed chemical composition of material used in this study

mol%	Na_2_O	K_2_O	Al_2_O_3_	SiO_2_	Eu_2_O_3_
LSX‐c	19	6	24	51	–
LSX	16	5	24	55	–
LSX:Eu	5	2	26	55	12

Commercial zeolite X (LSX‐c, Chemiewerk Bad Köstritz, Germany) was melted to form a reference melt‐quenched glass at 1650 °C, in a platinum crucible. The analyzed composition of this material corresponds closely to the synthesized LSX (Table 1).

For LSX:Eu^3+^, LSX was ion‐exchanged, filtered, washed, and dried at 60 °C. The analyzed composition is given in Table 1. Ion exchange involved immersing 2.8 g of LSX in a solution of 5 g of reagent‐grade EuCl_3_ and 160 mL of water (0.085 m) at 40 °C for 3 d. Due to the different chemical composition of LSX:Eu, the specific temperature range of melting deviates from those of the undoped LSX and LSX‐c, requiring different annealing protocols: (1) Low‐temperature samples were produced by heating between 75 and 600 °C, keeping the sample at the peak temperature for 1 h, from which the influence of water and further reactions which are not related to actual melting were elucidated; two other groups of samples were heated to (2) 830 and (3) 850 °C, respectively, and held at these temperatures for varying time intervals. The heating rate employed in all experiments was 10 K min^−1^.

All chemical analyses were done by inductively coupled plasma optical emission spectroscopy (ICP‐OES).

Beam bending viscometry was conducted on LSX‐c glass using samples with dimensions of 47 × 4 × 3 mm^3^, at a heating rate of 10 K min^−1^ in two separate scans at loads of 20 and 400 g (Figure S2, Supporting Information).

*Structural Characterization XRD*: High‐resolution powder synchrotron radiation diffraction was performed on the zeolite LSX, the reference glass, and on the collapsed HDA zeolite at DESY. The radiation wavelength (0.02080 nm) was calibrated from a CeO_2_ powder standard in capillaries and slab‐shaped samples. The 2D scattering patterns were integrated to functions of scattering angle (2Θ) and corrected for container scattering, absorption, and background.^41^ Intensities *I*_corr_(*Q*) were normalized according to the sample's chemical compositions using tabulated data^42^, ^43^ of the atomic coherent and Compton scattering. The X‐ray structure factor *S*(*Q*) was calculated as

(3) $$S(Q)\;=\;\frac{I_{\mathrm{corr}}(Q)\cdot N\;-\;\left\langle f^{2}(Q)\right\rangle \;-\;I_{\mathrm{Compton}}}{{\left\langle f(Q)\right\rangle }^{2}}$$

where 〈…〉 indicates the average sample composition, *f*(*Q*) is the coherent atomic scattering amplitude, and *N* the normalization factor. The mass density of the melt‐quenched glass was 2.49 g cm^−3^. By reference to a similar LSX20 crystal (ICSD 85621^44^) we estimated 8 H_2_O per formula unit for the zeolite, based upon its mass density of 2.06 g cm^−3^.

*XRD Collapse Measurements*: In situ collapse studies of LSX samples were performed in air using a Bruker D8 diffractometer equipped with an Anton Paar HTK1200 heating stage and a Vantec position‐sensitive detector that enables rapid data collection. A collimated spectrally pure Cu Kα X‐ray beam was produced using an incident beam Goebel mirror, enabling low backgrounds to below 3° 2Θ. Samples were mounted as thin layers of powder onto nonreactive sapphire crystal substrates. Time–temperature profiles for the stepwise measurement of reaction sequences included heating at 120 K min^−1^ to the target temperature of 725 °C, followed by subsequent ramping to higher temperatures at 20 K min^−1^. For the kinetic studies, a ramp rate of 120 K min^−1^ was used to the target temperature, at which point back‐to‐back XRD measurements were performed using time intervals from 15 to 120 s per XRD pattern. Data analysis was performed using Bruker's software Diffrac.EVA and Topas.

*Fluorescence Spectroscopy*: Room‐temperature fluorescence spectroscopy was employed to examine local structural symmetry. Static photoluminescence excitation (PLE) and emission spectra (PL) were recorded on compressed powders of 5 mm in diameter (produced by uniaxial compaction at 1 GPa), using a high‐resolution spectrofluorometer (Fluorolog 3, Horiba) with a continuous‐wave 450 W Xe lamp as excitation source, and a Hamamatsu R2658P photomultiplier tube (PMT) for detection. Corrections of the PLE spectra were performed over the lamp intensity, while PL spectra were corrected according to the spectral sensitivity of the employed PMT. Using the excitation line of ^7^F_0_→^5^L_6_ at the wavelength of 393 nm, the intensity ratio of the emission bands of ^5^D_0_→^7^F_2_ (≈612 nm) and ^5^D_0_→^7^F_1_ (≈591 nm) was determined as a measure of local ligand symmetry.^36^

*IXS and NMR Spectroscopy*: IXS experiments were performed at ID16 at the ESRF^45^ with a scattering wavevector (4π sin θ/λ) *Q* of 0.2 nm^−1^ (Figure S6, Supporting Information). Data were reduced combining the Rayleigh line, defined by the instrument function, with a symmetrical Brillouin doublet. The ratio of the two areas, the Landau–Placzek ration *R*_LP_, was used to obtain the nonergodicity factor *f*_0_ (Figure 2d). The longitudinal speed of sound (Figure S7, Supporting Information) was determined from *V*_L_ = ω_Q_/*Q* where *ω_Q_* is the inelastic frequency at *Q*.

^29^Si magic angle spinning NMR measurements (Figure S6, Supporting Information) were recorded on a Bruker Advance DSX 400 9.4 T spectrometer. Resonance frequencies of 79.5 MHz were used in a 7 mm magic angle spinning probe, with ZrO_2_ rotors at 5 kHz with a 1.4 µs (θ = π/3) pulse applied and a repetition time of 60 s. Chemical shifts were calibrated against trimethyl silane (Figure S6, Supporting Information).

*Densities*: Densities were obtained using pycnometer methods (Figure S8, Supporting Information) INS spectroscopy. Details of the application of INS in following the THz signatures of librational and zeolite subunit collective modes are given in ref. 46. These vibrational states persist over the course of the low‐temperature melting zeo‐LDA component. Moreover, removing the areas of the anharmonic contributions in proportion to the crystalline zeolite left reveals the early growth of librational modes coinciding with the topological invariant zeo‐LDA transition, followed by the gradual rise in the sodalite cage with its eventual decline, as the order–disorder LDA‐HDA transition is reached (Figure S9, Supporting Information).

*Reversibility of Decelerated Melting Transitions*: As it was necessary to retain specimens amorphized ex situ over several years—checking the degree of amorphization periodically—the gradual recovery of crystallinity was discovered retrospectively (Figure 2e). Amorphization (1 − *g*(*r*)) was conducted initially by progressively heating and holding for 2 h at 650 °C (25%), 795 °C (75%), and 850 °C (100%). The recovery of crystallinity over several years confirmed, for the first time, the reversibility of zeolite melting LSX‐LDA and the LDA‐HDA polyamorphic transition, viz., the transitions labeled (B) HDA‐LDA and (A) LDA‐LSX, identified from ^29^Si NMR and IXS in Figure 2c,d, and implicit in the model illustrated in Figure 1e,f and in the visualization of MD trajectories Vol 0 to Vol 11.

*Computational Details*: The faujasite structure comprises six‐membered double rings and sodalite β cages, from which α supercages are constructed (see also Figure 3c). The pure form of silicalite (SiO_2_) provides a fully cross‐linked model system for network investigations in the absence of mobile cations. MD simulations to complement the experimental observation of faujasite collapse with mechanistic insights on the molecular scale were used. This was done in a sequence of stepwise volume reduction during isothermal treatment at temperatures from 800 to 1200 K. The computational layout is depicted in Figure 3a. MD was performed using the large‐scale atomic/molecular massively parallel simulator (LAMMPS)^47^ with a reactive force field ReaxFF^48^, ^49^ and periodic boundary conditions on a (SiO_2_)_192_ cell. Cubic silicalite was first energetically optimized. Second, the simulation temperature was raised from 300 to 800, 900, 1000, 1100, or 1200 K, respectively, at a rate of 0.25 K ps^−1^ by employing the isothermal–isobaric (*NPT*) ensemble. Control of temperature and pressure was done by velocity scaling at every time step, using the Nosé–Hoover barostat with zero target pressure and the equations of motion of Shinoda et al.^50^, ^51^ After reaching the respective target temperature, the structure was again equilibrated over 300 ps using the Nosé–Hoover thermostat.^52^, ^53^ Next, the overall cell volume was scaled to match the average volume obtained from the preceding *NPT* simulation at the corresponding temperature (VOL0). Then, the volume was sequentially reduced in equivalent steps (Table S4, Supporting Information) such that VOL 10 exhibited the experimental mass density of vitreous silica. Between each step, the system was equilibrated for 1 ns by using the canonical (*NVT*) ensemble and the Nosé–Hoover thermostat. In rare cases, when convergence was not achieved within this time, the equilibration step was prolonged to 2 ns. Average properties were evaluated for the last 200 ps of each *NVT* simulation. All simulations were repeated once in order to verify reproducibility.

The major outputs of this procedure were structure trajectories for the different stages of silicalite collapse, calculated at time steps of 0.5 fs. The subsequent analysis used trajectories for every 0.5 ps.

## Conflict of Interest

The authors declare no conflict of interest.

## Supporting information

Supplementary
