# Supplementary material for: Kinetics of Decelerated Melting
Source: Adv Sci (Weinh). 2018 Mar 1;5(5):1700850. doi: 10.1002/advs.201700850 (PMC5979640; doi:10.1002/advs.201700850)
Supplement: Supplementary file 1 — Supplementary [file ADVS-5-1700850-s001.pdf]

Copyright WILEY-VCH Verlag GmbH & Co. KGaA, 69469 Weinheim, Germany, 2018.

Supporting Information

for *Adv. Sci.*, DOI: 10.1002/advs.201700850

Kinetics of Decelerated Melting

Lothar Wondraczek, Zhiwen Pan, Theresia Palenta, Andreas Erlebach, Scott T. Misture, Marek Sierka, Matthieu Micoulaut, Uwe Hoppe, Joachim Deubener, and G. Neville Greaves*

Supplementary Information

Kinetics of decelerated melting

Lothar Wondraczek[1,2,*], Zhiwen Pan[1,2], Theresia Palenta[1,2], Andreas Erlebach[1], Scott T. Misture[3], Marek Sierka[1], Matthieu Micoulaut[4], Uwe Hoppe[5], Joachim Deubener[6], G. Neville Greaves[7]

[1]*Otto Schott Institute of Materials Research, University of Jena, 07743 Jena, Germany*

[2]*Center of Energy and Environmental Chemistry, University of Jena, 07743 Jena, Germany*

[3]*Inamori School of Engineering, Alfred University, 14802 Alfred, NY, USA*

[4]*Physique Théorique de la Matière Condensée, Paris Sorbonne Universités – UPMC, 75252 Paris, France*

[5]*Institute of Physics, Rostock University, 18059 Rostock, Germany*

[6]*Institute of Non-Metallic Materials, Clausthal University of Technology, 38678 Clausthal-Zellerfeld, Germany*

[7]*Department of Materials Science, University of Cambridge, CB3 0FS Cambridge, United Kingdom*

1 Kinetic model for two-step melting

A useful way to detect KWW behavior is the linearization of Eq. 1,

$$\ln\left[-\ln\left(\frac{A(t)-A_\infty}{A_0-A_\infty}\right)\right]=\ln\left[-\ln\left(\varphi(t)\right)\right]=\beta\ln t-\beta\ln\tau \qquad \text{(SE2)}$$

which, once conveniently plotting (ln(-ln($\phi(t)$)) as a function of ln(t)) should yield a straight line with β as the angular coefficient.

For the reaction from LSX to liquid, we assume a model reaction path as depicted in **Figure S1**.

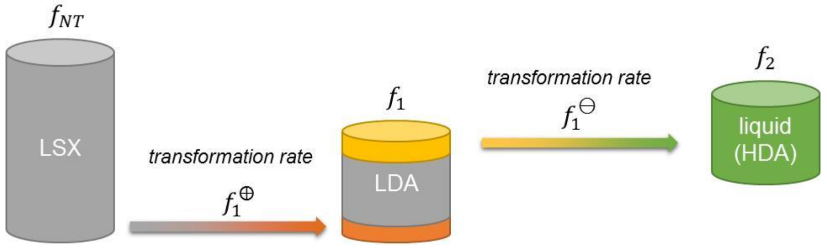

Fig. S1. Model reaction path for decelerated two-step melting.

Following a simple reaction rate scheme for sequential reactions, we have for the effective rate of LDA formation that

$$\frac{\partial f_1^{eff}(t)}{\partial t}=\frac{\partial f_1^{+}(t)}{\partial t}+\frac{\partial f_1^{-}(t)}{\partial t} \qquad \text{(SE3)}$$

, where $\frac{\partial f_1^{+}(t)}{\partial t}$ is the appearance rate of LDA due to the reaction LSX→LDA, and $\frac{\partial f_1^{-}(t)}{\partial t}$ is the disappearance rate of LDA due to the reaction of LDA→HDA.

From our hypotheses, we now assume that the non-transformed fraction of LSX follows a KWW dependence given by

$$f_{NT}(t)=\exp\left(-\left(\frac{t}{\tau_1}\right)^{\beta}\right) \qquad \text{(SE4)}$$

, therefore,

$$\frac{\partial f_1^+(t)}{\partial t} = -\frac{\partial f_{NT}}{\partial T} = \frac{\beta}{\tau_1}\left(\frac{t}{\tau_1}\right)^{\beta-1} \exp\left(-\left(\frac{t}{\tau_1}\right)^{\beta}\right) \text{ with } \beta \geq 1. \qquad \text{(SE5)}$$

We argue in the manuscript that $\beta = 2$ for the first reaction, and $\beta = 1$ for the second reaction. When solely the reaction of LDA→HDA is considered, it is further

$$f_1(t) = \exp\left(-\frac{t}{\tau_2}\right) \qquad \text{(SE6)}$$

and so

$$\frac{\partial f_1^-(t)}{\partial t} = -\frac{1}{\tau_2}\exp\left(-\frac{t}{\tau_2}\right). \qquad \text{(SE7)}$$

Eq. SE7 is the disappearance rate of LDA, not the effective (overall) rate. Substituting into Eq. (1) provided in the manuscript, we find that the effective rate of change of LDA is given by

$$\frac{df_1^{eff}(t)}{dt} = \left[\frac{\beta}{\tau_1}\left(\frac{t}{\tau_1}\right)^{\beta-1} \exp\left(-\left(\frac{t}{\tau_1}\right)^{\beta}\right)\right] + \left[-\frac{1}{\tau_2}\exp\left(-\frac{t}{\tau_2}\right)\right] \qquad \text{(SE8)}$$

, or, rearranging,

$$\frac{1}{\tau_2} f_1(t) + \frac{\partial f_1^{eff}(t)}{\partial t} = \frac{\beta}{\tau_1}\left(\frac{t}{\tau_1}\right)^{\beta-1} \exp\left(-\left(\frac{t}{\tau_1}\right)^{\beta}\right). \qquad \text{(SE9)}$$

Integrating Eq. SE9,

$$f_1(t) = \frac{\beta}{\tau_1^{\beta}} \exp\left(-\frac{t}{\tau_2}\right) \int t^{\beta-1} \exp\left(\frac{t}{\tau_2}\right) \exp\left(-\left(\frac{t}{\tau_1}\right)^{\beta}\right) dt. \qquad \text{(SE10)}$$

In the context of this study, a reasonable analytical solution of this integral is available only for the cases of $\beta = \{1,2\}$. In accordance with the previous arguments, here, we analyze the experimental data for $\beta = 2$. This yields the function for $g(t)$ as stated in Eq. 2. The extracted reaction timescales are provided in **Table S1**.

Table S1: Reaction times τ_1 and τ_2 as obtained from fitting the experimental data to Eq. 2. The effective reaction time τ_{eff} is read from the data shown in **Figure 1c** of the manuscript for φ = 1/e after linear interpolation between sequential data points.

temperature (°C)	τ_{eff} (± 10 s)	τ_1 (± 30 s)	τ_2 (± 30 s)
780	1895	653	1460
790	1420	683	773
798	1010	469	585
803	915	697	294
810	830	654	171
slope ln(τ/s) vs. T^{-1} (K)	33100	(881)	80464
Pearson-R	0.99	(0)	0.98

2 Analysis of reaction timescales

Fig. S2 depicts the viscosity curve from which Maxwell melt relaxation times as shown in **Fig. 1** were derived.

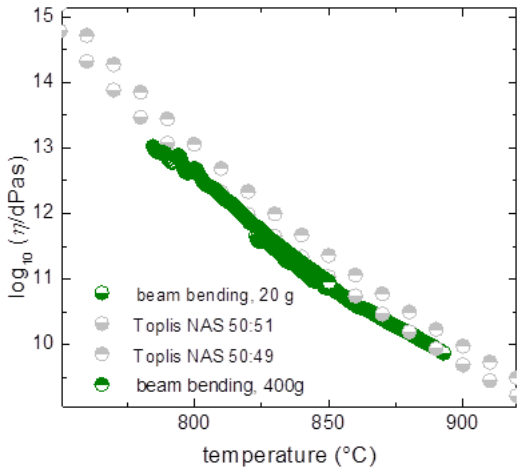

Fig. S2. Viscosity of melt-derived glass with LSX composition. For reference, data from Toplis *et al*. [1] for similar compositions are also shown.

The temperature-dependence of the reaction times as extracted from fitting the XRD data to Eq. 2 (**Table S2**) of the manuscript was framed within a simple Arrhenius equation,

$$\tau_{1,2,eff}(T) = \tau_0 \exp\left(-\frac{\Delta H}{\mathrm{R}T}\right) \quad \text{(SE11)}$$

, with intercept τ_0, energy barrier ΔH and the universal gas constant R. This assumes constant ΔH in all three cases. For τ_1, this is taken as resulting from the super-strong nature of the LSX → LDA transition [2]. For τ_2 and τ_{eff}, it is a reasonable approximation within the relatively narrow viscosity range of consideration [3]. The resulting Arrhenius plots are provided in **Figure 1e** of the manuscript. Slopes of ln(τ) vs. 1/T are given in **Table S2**.

In order to judge the observed timescale, classical data of surface pre-melting was considered. Due to the lack of data on carnegieite, albite was used for reference. Albite pre-melting has been described by Greenwood and Hess [4]. In their report, surface melting rates are provided for a range of temperatures close to the melting temperature of albite, *i.e.*, 1120 °C. In order to approximate reaction times from these melting rates, we calculated the melting progress of a spherical particle with diameter *d*, and extracted the time τ_{SPM} after which the melted volume has reached a fraction of (1-1/e) as shown in **Figure S3**. This situation best resembles the experimental set-up shown in **Figure 1**. In particular, it avoids the notorious divergence of other approaches for translating reaction rates into characteristic times such as the Stokes-Einstein equation.

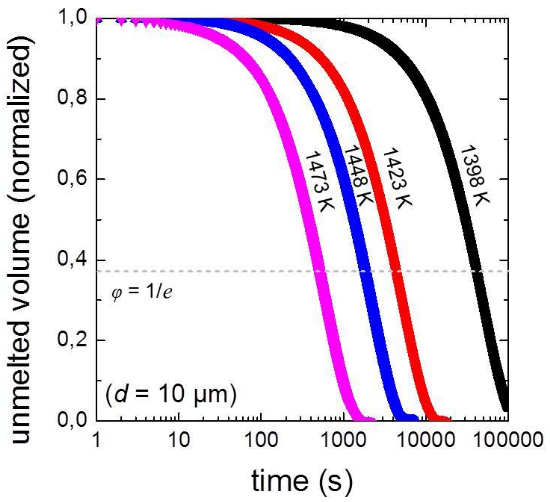

Fig. S3. Surface melting progress of crystalline albite for spherical particles with diameter of 10 µm. Rates according to [4].

3 Reproducibility of isothermal experiments

The following graph gives an example of reproduction. Here, the measurement at 798 °C was conducted two times, on individual samples, using different X-ray detection angular bandwidth for differently fast data acquisition.

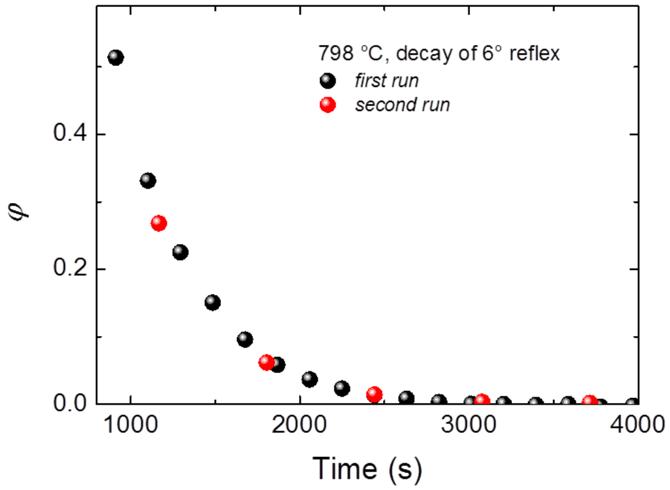

Fig. S4. Reproducibility of successive collapse runs of LSX using XRD.

4 Photoluminescence probing

Progress of XRD and PL emission spectra with annealing for LSX:Eu at 830 °C and 850 °C is shown in Fig. S5. Asymmetry data is shown in Fig. 2b.

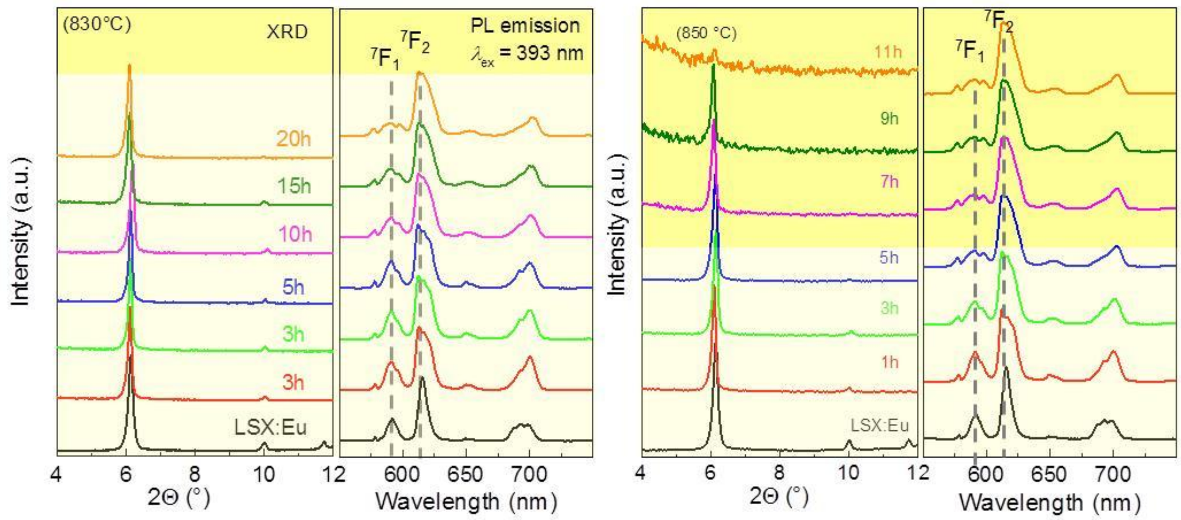

Fig. S5. Development of XRD Eu^{3+} LSX from with annealing.

5 NMR and IXS Analysis

NMR and IXS data are provided in the following Figs. S6-8.

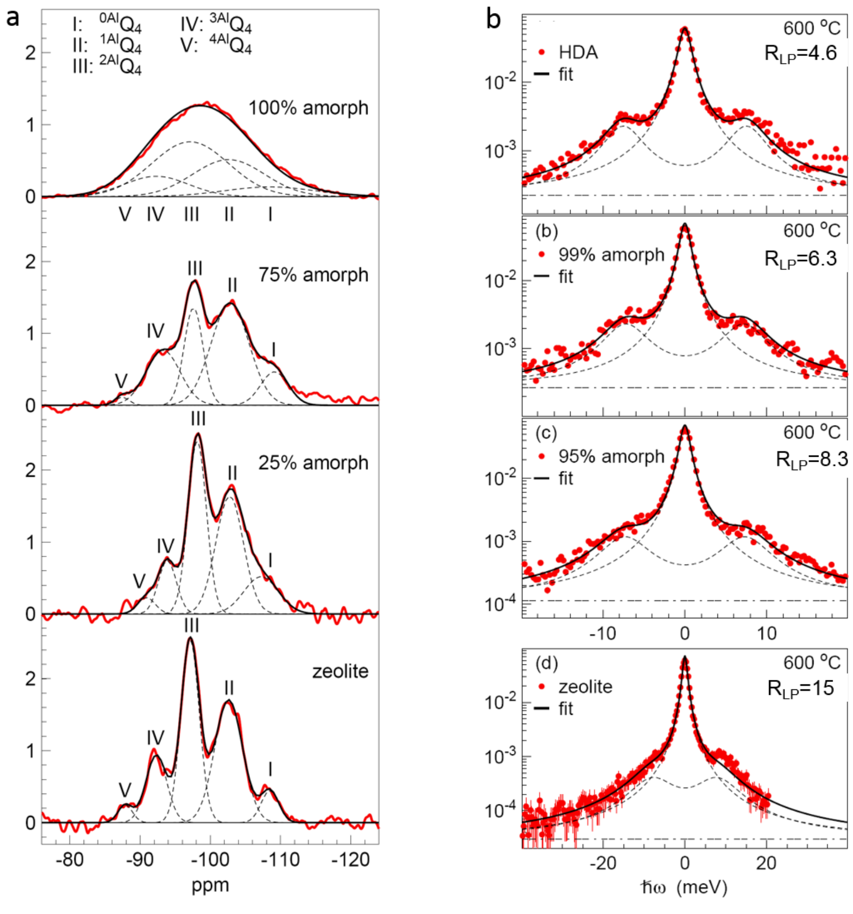

Fig. S6. *Ex situ* NMR spectra of Na zeolite Y showing Al-Si Q species I ($^{0A}lQ_4$), II ($^{1Al}Q_4$), III ($^{2Al}Q_4$), IV ($^{3Al}Q_4$) and V ($^{4Al}Q_4$) (left), and IXS spectra showing elastic Rayleigh peak and inelastic Brillouin doublet (right) - each at different stages of amorphization.

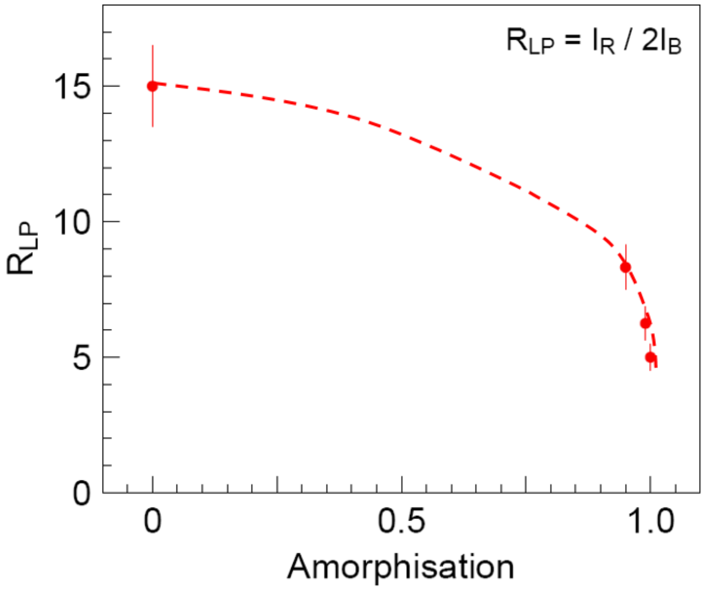

Fig. S7. Landau-Placzek ratio RLP analyzed from IXS spectra in Fig. S6. Arrows indicate transitions zeo-LDA (A) and LDA-HDA (B) obtained from Fig. 2c.

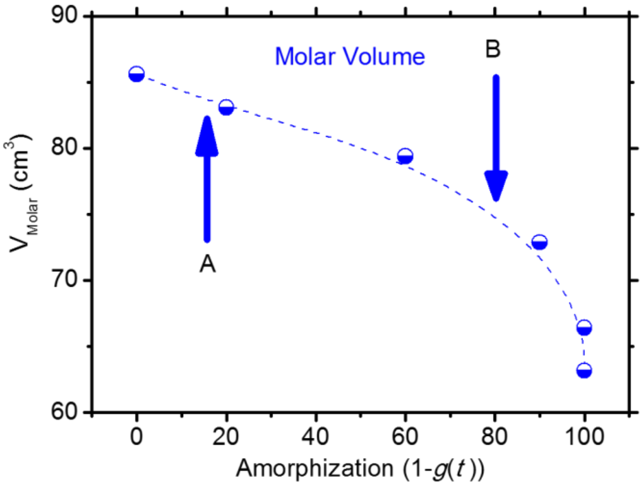

Fig. S8. Reduction in molar volume with increased amophization of Na zeolite Y. Arrows indicate transitions zeo-LDA (A) and LDA-HDA (B) obtained from Fig. 2c.

6 INS Analysis

INS data are provided in Fig. S9.

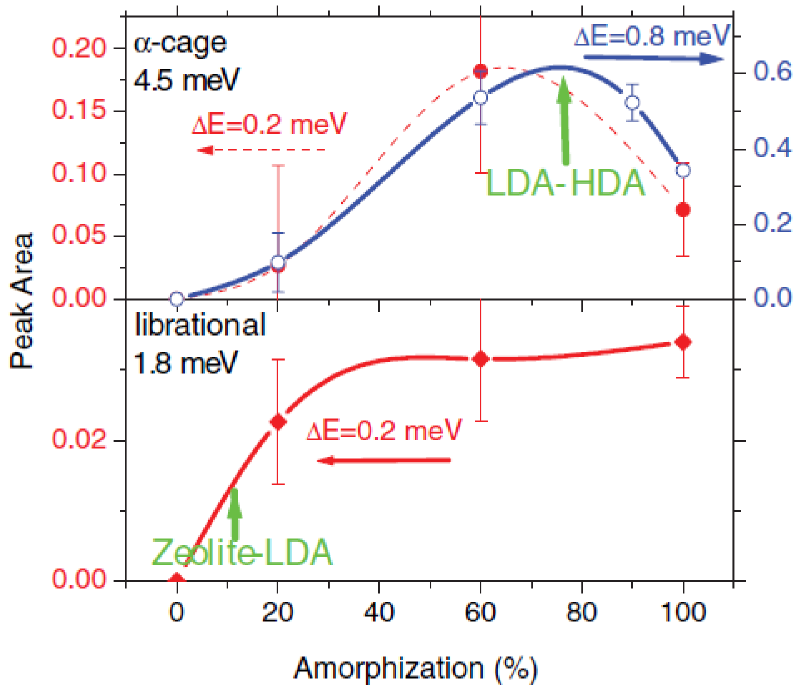

Fig. S9. Anharmonic contributions to amorphized content obtained using inelastic neutron scattering [**Error! Bookmark not defined.**], showing early growth of librational modes coinciding with topological invariant zeo-LDA transition, followed by gradual rise in the sodalite cage with its eventual decline as the order-disorder LDA-HDA transition is reached.

7 Reproducibility of MD simulation

The following figure shows a reproduction of MD simulations.

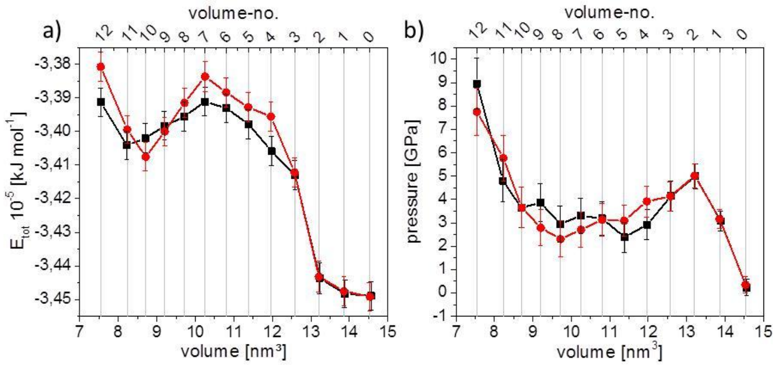

Fig. S10. Development of the total energy (a) and the internal pressure (b) of two simulations carried out at T = 1200 K.

8 Structural equivalence of collapsed LSX (HDA) and melt-derived glass

Figure 2a of the manuscript shows the structure factors of the LSX crystalline material, the collapsed LSX and the melt-quenched glass, all three measured as powders inside capillaries. The LSX crystal shows the typical powder diagram of a crystalline material but the Bragg reflections appear poorly resolved. The detector setting is optimized for covering a large range of scattering angles but that is accompanied with a poor angular resolution. The $S(Q)$ of the collapsed LSX shows an amorphous structure with only tiny relicts of the LSX

crystal. **Fig. S11** compares the S(Q)'s of the LSX collapsed and the LSX glass. The differences are very small. Moreover, the LSX glass was measured in capillary and as slab-shaped sample. Both these S(Q)'s are nearly identical. The drop of intensities close to 200 nm^{-1} for all three *S*(*Q*)'s seems to have an unphysical origin.

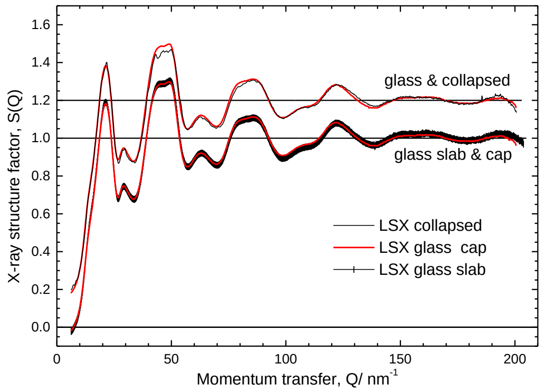

Fig. S11. Comparison of the melt-derived glass and collapsed LSX as measured in capillary or as plate. The upper functions are shifted vertically for clarity.

The real-space correlation functions, *T*(*r*), were calculated as Fourier transforms (FT) of the *S*(*Q*)'s with Q_{max} = 200 nm^{-1} through

$$T(r) = 4\pi\, r\, \rho_0 + 2/\pi \int_0^{Q_{max}} Q\,[S(Q)-1]\,M(Q)\,\sin(Qr)\,\mathrm{d}Q \qquad \text{(S1)}$$

, either using the damping factor *M*(*Q*) according to Lorch [5] or without damping [*M*(*Q*) = 1]. The number densities of atoms, ρ_0, are calculated from the mass densities. The resulting *T*(*r*) curves obtained with damping are compared in the manuscript **Figure 2b** up to distances of 1.0 nm. The *T*(*r*) curve of the LSX crystal shows clear peaks throughout the full range whereas the other two *T*(*r*)'s are rather smooth for $r > 0.7$ nm. The first peaks up to 0.32 nm are in equal positions for LSX glass and crystal but the peaks at larger distances differ in their positions. On the other hand, the *T*(*r*)'s of the collapsed LSX and of the glass are visibly identical. Features in this distance determine the positions of the first sharp diffraction peaks (FSDP). Surprisingly, the FSDP's of LSX glass and collapsed LSX do not differ significantly in the height or width. Thus, the amount of structural defects of the collapsed LSX is not sufficient to produce significant changes of the medium-range order as compared to the melt-quenched glass.

The *T*(*r*) data of all four samples are shown in **Fig. S12** and compared with the model *T*(*r*) functions. The fits were performed in a distance range between 0.12 nm and 0.26 nm for the *T*(*r*)'s obtained with damping. The curves appear smooth in this range except of the first peak at ~0.17 nm that is due to Si-O and Al-O bonds. All distances between the lengths of the Al-O bonds and the Si(Al)-Si distances at 0.31 nm are attributable to the Na-O and O-O partial correlations. The shortest O-O distances are the tetrahedral edges of the SiO_4 at 0.265 nm and the AlO_4 at 0.280 nm. The O-O edges of possible AlO_6 octahedra would have lengths of 0.253 nm. The number of all these edges is simply calculated according to the amount of the corresponding structural units.

The *T*(*r*) curve of the sample LSX crystal needs additional distances for its simulation. The water filling the holes of LSX shows typical O-O and O-H distances. Water forms a tetrahedral environment of H_2O molecules, i.e. each O has four O neighbors, two short and

two long O-H bonds exist for each O that are characteristic for O-H ... O hydrogen bridges. The corresponding coordination numbers given are reduced because only half of the O atoms belong to the fraction of water. The surface of the alumosilicate backbone participates in the structure of the water via hydrogen bridges.

The *T*(*r*) curves obtained without damping show sharp peaks, among them also new features at 0.21 nm and 0.24 nm that could be related to the Na-O distances expected for these lengths. It is shown below that the satellite oscillations of the narrow Si-O and O-O peaks contribute to the neighboring peaks and direct estimations of the Na-O coordination numbers from the small peaks are misleading.

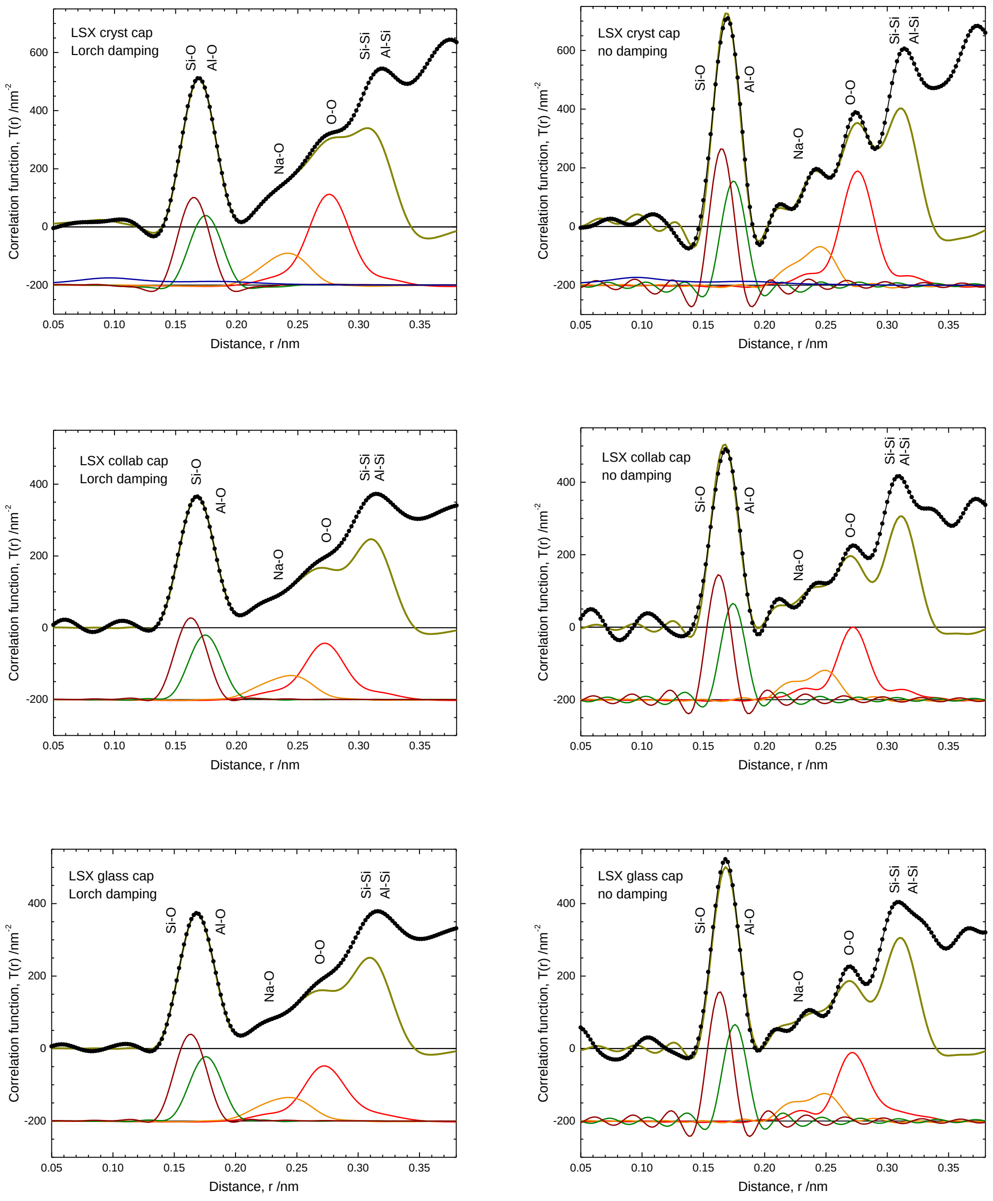

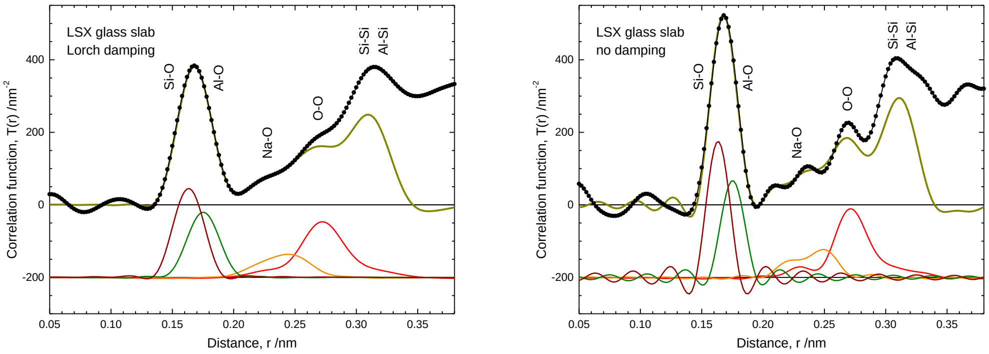

Fig. S12. Correlation functions with and without damping – experimental (dotted lines) and model (thick lines) data of four samples of LSX. The fits were performed with the experimental T(r) with Lorch damping. The partial correlation functions of the Si-O, Al-O, Na-O and O-O pairs are shifted downwards for better visibility. The smooth correlation O-H with distances at 0.10 nm and 0.18 nm is shown for the LSX crystal. It might be worth extending these partials and total G(r) to include the split peak for 4 and 6 fold rings 3.8-4.4 Å in LSX and in simulated LSX-LDA transition in silicalite.

The parameters of the Al-O and Na-O first-neighbor distances are determined by Gaussian fitting of the smooth $T(r)$ functions that were obtained with damping. The effects of damping and truncation (Q_{max}) in the FT of the experimental $T(r)$ data are simulated for the model $T(r)$ functions by the convolution techniques as described in [6,7]. For fitting the $T(r)$ data, the Marquardt algorithm [8] is used where coordination numbers, N_{ij}, mean distances, r_{ij}, and full widths at half maximum (fwhm), Δr_{ij}, are the parameters of the model Gaussian functions. The final parameters are listed in **Table S3** where each line corresponds to a Gaussian function. Some parameters are marked with asterisks in case of the first sample. The corresponding parameters were fixed in the fits of this and the other samples. Since Al-O and Na-O distances overlap with the neighboring Si-O and O-O peaks several assumptions for the parameters are needed to obtain useful results. The lengths of the Al-O bonds can range from 0.170 nm to 0.190 nm. The known AlO_x groups are the AlO_4 tetrahedron, AlO_5 pyramid and AlO_6 octahedron. However, only AlO_4 groups exist in the similar LSX20 crystal [**Error! Bookmark not defined.**] and this is expected also for the glass. Except of the expected AlO_4 only a small fraction of AlO_6 is assumed. If the latter fraction is smaller than the uncertainty of the coordination number one should not interpret these larger units as real entities.

The Al-O bonds of AlO_4 or AlO_6 overlap with the Si-O peak (~0.162 nm) of the SiO_4 unit. Due to the smaller Al_2O_3 content if compared with that of SiO_2 the fraction of AlO_6 units will possess larger uncertainty. N_{SiO} was fixed to 4.0 in the fits because simultaneous determinations of the values N_{SiO} and N_{AlO} are not possible. The longer Al-O bonds overlap only partially with the Na-O peaks. The starting parameters of the peaks were taken from the related crystal structure of LSX20 [**Error! Bookmark not defined.**].

For illustration of the quality of the fits also those model $T(r)$ functions are calculated and compared with the experimental data which were obtained without use of damping functions in FT (Fig. 4). Here, the agreement is excellent for the LSX crystal, as well, just not perfect for the other samples. Distinct peaks are visible at 0.21 nm and 0.24 nm which could be

related to the Na-O bonds. The partial $T_{ij}(r)$ functions, they are also shown in the plots, illustrate the effects of the truncation oscillations of the Fourier integral. Considerable contributions to the visible peaks at 0.21 nm and 0.24 nm are due to the truncation oscillations of the Si-O and O-O correlations.

The results of the fits do not show any new sensations or discoveries but they follow the expectations for the samples and confirm the usefulness of the assumptions. The N_{AlO} values are a little larger than four but that is not sufficient to conclude a significant AlO_6 fraction. The N_{NaO} value of the LSX crystal is close to six which is significantly larger than the other N_{NaO} of the collapsed LSX or the LSX glass. The larger N_{NaO} could be attributed to the crystalline order and the presence of water.

Table S2. Parameters of the Gaussian functions used or obtained in the fits of the $T(r)$ functions of three LSX samples in capillaries and a slab-shaped LSX glass. The parameters marked with asterisks for sample LSX crystal were fixed or calculated throughout all samples.

	**i - j**	N_{ij}	r_{ij}	Δr_{ij}	**total** N_{ij}	**mean bond length i - j**
LSX crystal	Si-O	4.00*	0.1649	0.013	4.0	0.1649±0.0015
	Al-O	3.50	0.1736	0.015	4.25±0.30	0.175±0.002
		0.75	0.1802	0.016		
	Na-O	1.80	0.2235*	0.018*	5.7±0.5	0.239±0.003
		3.90	0.2465	0.020*		
	K-O	2.00*	0.2950*	0.025*	4.0	
		2.00*	0.3100*	0.020*		
	O-O	1.58*	0.2680*	0.018*		SiO_4 edges
		1.26*	0.2810*	0.022*		AlO_4 edges
		0.36*	0.2530*	0.020*		AlO_6 edges
		2.00*	0.2820*	0.025*		H_2O structure
	Al-Si	4.00*	0.3135*	0.025*	4.0	
	O-H	1.00*	0.0950*	0.015*	2.0	H_2O structure
		1.00*	0.1880*	0.030*		
collapsed LSX (HDA)	Si-O	4.00	0.1625	0.0145	4.0	0.1625±0.0015
	Al-O	3.60	0.1735	0.015	4.2±0.3	0.174±0.002
		0.60	0.1800	0.017		
	Na-O	1.60	0.2235	0.018	4.8±0.5	0.241±0.003
		3.20	0.2490	0.020		
	K-O	2.00	0.2950	0.025	4.0	
		2.00	0.3100	0.020		
	O-O	3.03	0.2680	0.018		SiO_4 edges
		2.48	0.2810	0.020		AlO_4 edges
		0.55	0.2531	0.020		AlO_6 edges
	Al-Si	4.00	0.3135	0.025	4.0	
melt-quenched glass (capillary)	Si-O	4.00	0.1633	0.014	4.0	0.1633±0.0015
	Al-O	3.80	0.1755	0.0145	4.1±0.3	0.176±0.002

		0.30	0.1800	0.016		
	Na-O	1.70	0.2240	0.018	4.7±0.5	0.240±0.003
		3.00	0.2490	0.020		
	K-O	2.00	0.2950	0.025	4.0	
		2.00	0.3100	0.020		
	O-O	3.03	0.2670	0.018		SiO_4 edges
		2.76	0.2825	0.022		AlO_4 edges
		0.28	0.2531	0.020		AlO_6 edges
	Al-Si	4.00	0.3135	0.025	4.0	
melt-quenched glass (slab)	Si-O	4.00	0.1633	0.0135	4.0	0.1633±0.0015
	Al-O	3.80	0.1750	0.015	4.10±0.30	0.175±0.002
		0.30	0.1780	0.017		
	Na-O	1.50	0.2240	0.018	4.5±0.5	0.241±0.003
		3.00	0.2490	0.020		
	K-O	2.00	0.2950	0.025	4.0	
		2.00	0.3100	0.025		
	O-O	3.03	0.2670	0.018		SiO_4 edges
		2.76	0.2825	0.022		AlO_4 edges
		0.28	0.2531	0.020		AlO_6 edges
	Al-Si	4.00	0.3135	0.025	4.0	

9 Computational details

Cell parameters for MD simulation:

Table S3. Mass density ρ, cell parameter *a* and volume *V* of cubic unit cells **VOL0** – **VOL12** (**VOL0** refers to T = 1200 K).

	ρ [g/cm^3]	*a* [Å]	*V* [nm^3]
**VOL0**	1.315	24.420	14.563
**VOL1**	1.380	24.035	13.884
**VOL2**	1.448	23.649	13.226
**VOL3**	1.522	23.263	12.590
**VOL4**	1.600	22.878	11.974
**VOL5**	1.683	22.492	11.379
**VOL6**	1.773	22.107	10.803
**VOL7**	1.869	21.721	10.248

**VOL8**	1.972	21.335	9.712
**VOL9**	2.083	20.950	9.195
**VOL10**	2.202	20.564	8.696
**VOL11**	2.330	20.179	8.216
**VOL12**	2.540	19.611	7.542

10 References

[1] M. J. Toplis, D. B. Dingwell, K.-U. Hess, T. Lenci, *Am. Mineral.* **82**, 979-990 (1997).

[2] G. N. Greaves, F. Meneau, A. Sapelkin, L. M. Colyer, I. A. Gwynn, S. Wade, G. Sankar, *Nat. Mater.* **2**, 622-629 (2003).

[3] C.A. Angell, *Science* **267**, 1924-1935 (1995).

[4] J. P. Greenwood, P. C. Hess, *J. Geophys. Res.* **103**, 29815-29828 (1998).

[5] E.A. Lorch, *J. Phys. C* **2**, 229 (1969).

[6] A.J. Leadbetter, A.C. Wright, *J. Non-Cryst. Solids* **7**, 23 (1972).

[7] R.L. Mozzi, B.E. Warren, *J. Appl. Crystallogr.* **2**, 164 (1969).

[8] D. Marquardt, *SIAM J. Appl. Math.* **11**, 431 (1963).
